# Full-Cut Manufacture of Skin-Interfaced Microfluidic Patch with Copper Electrode for In Situ Admittance Sensing of Sweat Rate

**DOI:** 10.3390/bios13010067

**Published:** 2022-12-31

**Authors:** Lei Wei, Yuxin He, Zihan Lv, Daoyou Guo, Lin Cheng, Huaping Wu, Aiping Liu

**Affiliations:** 1 Key Laboratory of Optical Field Manipulation of Zhejiang Province, Zhejiang Sci-Tech University, Hangzhou 310018, China; 2 School of Physics and Electronics Engineering, Fuyang Normal University, Fuyang 236037, China; 3 Key Laboratory of Special Purpose Equipment and Advanced Processing Technology, Ministry of Education and Zhejiang Province, College of Mechanical Engineering, Zhejiang University of Technology, Hangzhou 310023, China

**Keywords:** sweat rate, microfluidic patch, copper electrode, laser cutting, admittance

## Abstract

Sweat-rate measurement has received more and more attention, especially for specific groups, such as athletes, soldiers and manual workers, due to their excessive sweat loss under prolonged intense heat stress, which increases the risk of dehydration and electrolyte imbalance. The highly effective manufacture of a sweat-sensing device is essential to its wide range of applications in perspiration-related physiological information detection. In this work, we propose a simple and cost-effective strategy for the manufacture of a microfluidic sweat-rate-sensing patch via laser cutting and transfer printing technology. A copper foil tape is used as the electrode for in situ admittance based sweat-rate-sensing. The detection circuits and measurement conditions are optimized to prevent the negative effect of an electrochemical reaction between a copper electrode and sweat for precise admittance measurement. In vitro and on-body experiments demonstrate that the copper electrode is applicable for admittance-based sweat sensing and is capable of achieving equivalent sensing accuracy as a gold electrode and that the proposed sensor structure can perform consecutive and accurate sweat-rate-sensing and facilitates a significant increase in manufacturing efficiency.

## 1. Introduction

Perspiration is a crucial role in the thermoregulation of the body. For specific groups, such as athletes, soldiers and manual workers, excessive sweat loss under prolonged intense heat stress may increase the risk of dehydration and electrolyte imbalance, which can degrade individuals’ physical performances, compromise their physiological status, and even develop into fatal heat-related illnesses [1,2]. Measurement of sweat rate is helpful to provide a reasonable guidance for fluid intake [3,4] to ensure water and electrolyte balance in vivo. Recently, colorimetric [5,6,7,8,9,10], volumetric [11,12], calorimetric [13], capacitive [14,15], and impedance (or admittance) [16,17,18,19,20,21] sensing methods have been developed for the in situ measurement of sweat rate, but several challenges remain, such as inconvenience in automatic monitoring [5,6,7,8,9,11], requiring precise alignment in the micromachining process [13,16,17,18], and the required pre-calibration [17]. Besides, the vacuum-evaporated gold electrode commonly used for sweat rates requires time-consuming, multistep micromachining processes and increases manufacturing costs accordingly. [16,17,20,22]. In contrast, a copper electrode is a simple and low-cost alternative, especially in small-batch production, and it has been used in water oxidation reaction and CO_2_ electroreduction [23,24]. However, when the copper electrode is immersed in sweat, the electrochemical reactions at the Cu/sweat interface may occur and lead to the dissolution of the copper electrode [25] or the generation of bubbles, which will seriously affect the measurement accuracy. In order to avoid the occurrence of electrochemical reactions, a non-contact measurement method for sweat rate based on the copper capacitive sensor was designed, but only a weak output signal could be obtained [14]. However, the acquisition of a strong, stable and veritable test signal of sweat rate based on the copper electrode sweat sensing remains challenging.

Herein, we report a microfluidic patch with a copper electrode to measure the sweat rate by calculating the increasing rate of the measured admittance of the electrode in a microchannel when sweat advanced in the microchannel. The electrode admittance was measured by detection circuits that were optimized to prevent the negative effect of the electrochemical reaction in contacting admittance measurement, namely filtering the DC component of the excitation signal passing through the sensor to avoid the DC polarization of the electrodes, setting the highest frequency and the lowest amplitude of the excitation signal to minimize the influence of the electrochemical reaction on admittance measurement. Furthermore, the special structural design of electrodes in the microchannel and the use of copper foil as electrode material enable the rapid manufacture of the entire microfluidic patch using only laser cutting and rapid transfer printing technologies without precise alignment operation, surface treatment or modification of electrodes. The proposed easy-to-manufacture and low-cost microfluidic patch is capable of outputting a strong and credible admittance signal. In vitro and on-body experiments together demonstrated the validity of using copper electrodes and verified the accuracy of the proposed microfluidic patch for sweat-rate measurement.

## 2. Materials and Methods

### 2.1. Structure Design of the Microfluidic Patch

**Figure 1:**
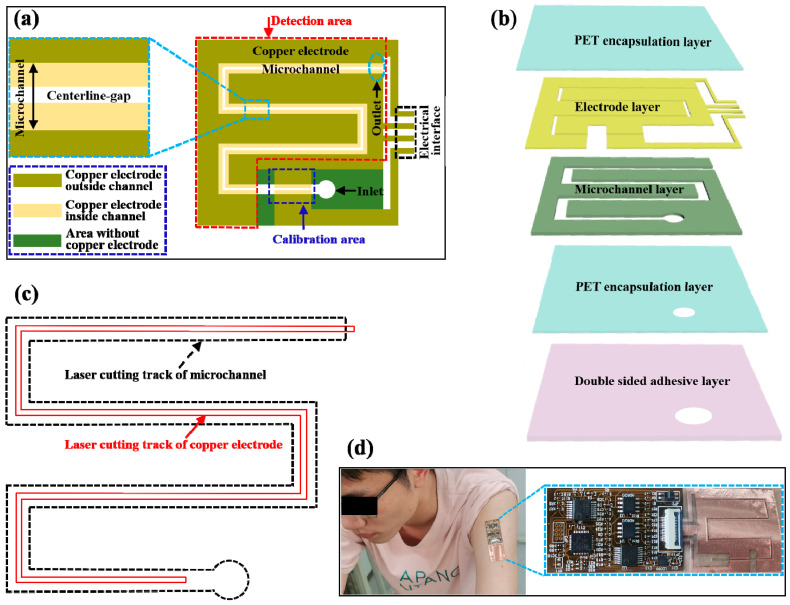
Design of microfluidic patch for sweat-rate sensing. (**a**) Structure of the microfluidic patch. (**b**) Exploded view of the patch. (**c**) Cutting tracks of microchannel and electrode. (**d**) Photograph of the patch integrated with FPCB mounted on the skin.

The microfluidic patch for sweat-rate measurement consists of three parts, namely detection area, calibration area (for sweat-rate calibration), and electrical interface, as illustrated in Figure 1a. The detection area and calibration area are constituted by a stacking microchannel layer and a copper electrode layer consisting of two pairs of parallel electrodes (Figure 1b). Only a small part of each electrode is located inside the microchannel, and every pair of the electrodes is separated by a distance of 200 μm (Figure 1a and Figure S1). Two PET films encapsulate the electrode layer and the microchannel layer. The microfluidic patch can be attached to the skin via a double-sided adhesive layer with a 5 mm hole for dynamic sweat sampling (Figure 1b). Collected sweat first flows into the 7.5 mm calibration area, then passes through it and enters the detection area. Newly-arrived sweat in the detection area increases the electrode admittance whose increment rate depends on the sweat rate and electrolyte concentration. The calibration area can decode the electrolyte concentration that is used to calibrate the sweat rate measured from the admittance of the detection area.

Based on the structure design above, the electrodes and microchannel can be conveniently fabricated by laser cutting, respectively following the corresponding cutting tracks shown in Figure 1c. Thus there is no need to embed two parallel copper electrodes into the microchannel by precise alignment operation, which can significantly improve manufacturing efficiency and product consistency. The patch integrated with a flexible printed circuit board (FPCB) can be easily mounted on the skin for in situ sweat collection and detection (Figure 1d).

It is important to note that, for sweat-rate measurement devices, the additional flow resistance of the device may affect the accuracy of sweat-rate detection. This is due to sweat being generated at the secretion coil of the eccrine gland and transported to the skin surface via the dermal duct and the upper coiled duct of the gland driven by secretory pressure [26,27], and the flow resistance of the gland and the secretory pressure determine the natural outflow rate of sweat from the sweat gland, which is the local sweat rate to be measured. However, a microfluidic sweat collection device inevitably increases the flow resistance in the sweat transport path. If the external flow resistance is large enough to be close to the sweat gland’s flow resistance, the sweat collection device will produce significant effect on the natural outflow rate of sweat from the sweat gland.

The flow of sweat in the microfluidic device is affected by both channel resistance and capillary pressure. Due to the small size of the microchannel, the microfluidic device may produce a considerable flow hindrance effect on sweat. In order to research the comprehensive impact of channel flow resistance and capillary pressure under a certain size of the microchannel, we study the equivalent flow resistance of the microchannel and obtain its formula as (more details refer to PART III of the Supplementary Materials):

(1) $$R_{eq}=\frac{P_{gland}R_{c}+\Delta P_{\sigma }R_{gland}}{P_{gland}-\Delta P_{\sigma }}$$

where *P*_gland_ is the sweat-gland secretion pressure, Δ*P*_σ_ is the capillary pressure, *R*_c_ is the flow resistance of the microchannel, and *R*_gland_ is the flow resistance of the gland.

In this work, the microchannel is produced by laser-cutting acrylic double-sided tape and is packaged with PET film. The contact angle of sweat with the tape and the PET film are 100° [28] and 80.5° [29], respectively. The study reported in Reference [14] also used the same material for microchannel fabrication with a channel size of 90 μm in height, 1 mm in width and 350 mm in length. The equivalent flow resistance they provided was 4.6 kPa·s·mm^−3^, consistent with the value of 4.68 kPa·s·mm^−3^ calculated by Formula (1), demonstrating the validity of Formula (1). For the channel size in this work (300 μm in height, 1 mm in width and 105 mm in length), the equivalent flow resistance of the microchannel is 0.31 kPa·s·mm^−3^, far less than the flow resistance of the gland (410 kPa·s·mm^−3^), so the designed microchannel device has little effect on the natural sweat secretion rate and sweat rate.

### 2.2. Principle of Admittance-Based Sweat-Rate Measurement via the Microfluidic Patch

**Figure 2:**
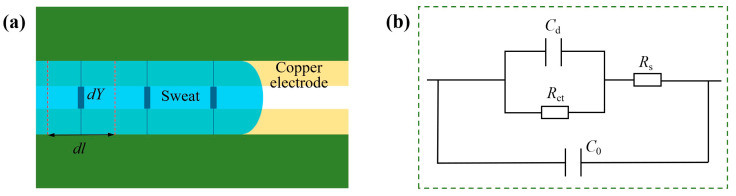
(**a**) Electrode admittance analysis model of sweat–electrode microsystem. (**b**) The equivalent circuit model of the microcell composed of electrodes and micro-sweat of length *dl*.

Sweat contains a large amount of electrolytes, such as Na^+^, Cl^−^ and K^+^, which endow sweat with certain conductivity. When a certain volume of sweat fills the detection area of length *dl* during the time from *t* to *t* + *dt* (Figure 2a), sweat and electrodes in this area of length *dl* will form a microcell whose equivalent circuit model is shown as Figure 2b, where *C*_d_ and *R*_ct_ represent the double-layer capacitance and charge-transfer resistance at the interface of the sweat-electrode, respectively. *R*_s_ is the solution resistance, and *C*_0_ is the distributed capacitance between two parallel electrodes.

The equivalent circuit in Figure 2b can be further simplified as the admittance *dY*. Considering that flowing sweat in the microchannel can be regarded as a series of parallel admittances between two electrodes (Figure 2a), the electrode admittance increases with the increase in sweat volume in the microchannel. Furthermore, *dY* is related to the sweat electrolyte concentration, so the flow rate of sweat in the microchannel can be calculated by the formula:

(2) $$Q_{i}=\frac{K}{c_{i}}\cdot \frac{Y_{i}-Y_{j}}{t_{i}-t_{j}}$$

where *c*_i_, and *Y*_i_ are the sweat electrolyte concentration and the electrode admittance of the detection area at moment *t*_i_, respectively, *t*_i_ and *t*_j_ are two adjacent sampling moments, and coefficient *K* is determined by both the microchannel size and the layout of electrodes in the channel. It is a constant for a specific sensor. The formula derivation refers to Part II of the Supplementary Materials.

### 2.3. Fabrication of Microfluidic Patch

**Figure 3:**
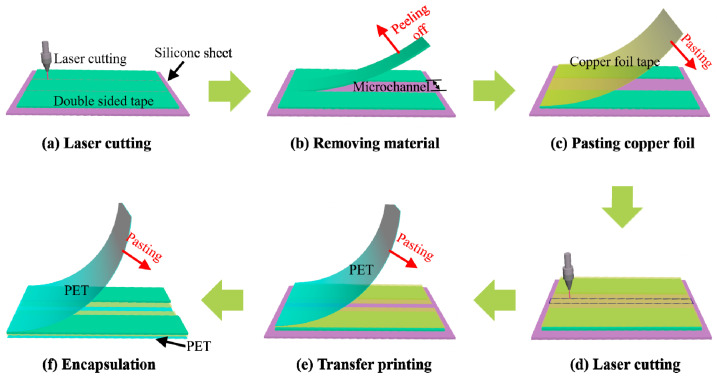
Fabrication process of microfluidic patch for admittance-based sensing of sweat rate.

A UV laser-cutter machine (ultraviolet laser-cutting machine-3W, Shenzhen Chaoyue Laser Intelligent Equipment Co., Ltd., ShenZhen, China) was used to fabricate all the layers of the microfluidic patch. The fabrication method and process are shown in Figure 3. First, the microchannel was fabricated by laser-cutting double-sided tape (9495LE 300LSE, 3M, Saint Paul, MN, USA) pasted on a silicone sheet pre-fixed on a countertop (Figure 3a) and then was peeled off the redundant area (Figure 3b). Then, copper foil tape (double conductive copper foil tape, Shenzhen Baojiasheng Adhesive Tape Products Factory, ShenZhen, China) was pasted onto the fixed microchannel layer and let its viscous surface be exposed (Figure 3c). After that, the UV laser was applied to cut copper foil along specific routes defined in the control software of the laser cutter machine (Figure 1c and Figure 3d). After peeling off the removable copper foil, a centerline-gap appeared, which divided the copper foil tape into two electrodes (Figure S1 and Figure 3d). Then a PET film (12 μm in thickness) was pasted on the adhesive surface of the copper foil to form strong interface adhesion between the PET film and the adhesive copper foil (Figure 3e). Thus, the microchannel and electrode layer could be transfer-printed onto the PET film by peeling them off together from the silicone sheet. With the help of the adhesion of the copper foil tape, the aligned electrode and microchannel could be rapidly transfer-printed onto PET film without the thermal release tape or the water soluble tape, so it is efficient and low-cost when compared to previously reported transfer-printing strategies [30,31]. At last, the microchannel was completely encapsulated with another PET film to form the microchannel patch (Figure 3f). The prepared patch was further integrated with a FPCB for in situ sweat-rate sensing (Figure 1d).

### 2.4. Building Experimental Testing System

**Figure 4:**
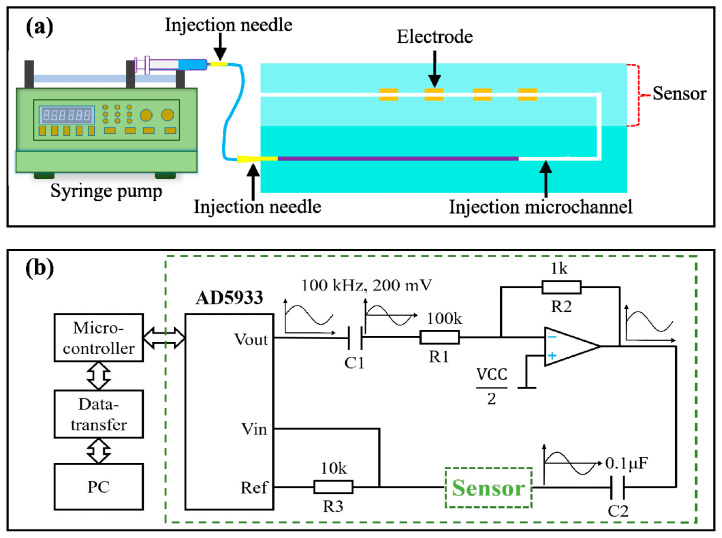
Schematic diagram of injection and measurement system. (**a**) Injection scheme of test solution in vitro experiments. (**b**) The schematic diagram of admittance measurement and data transmission.

The in vitro characterization of the sensing performance of the microfluidic patch was carried out by a self-built injection analysis system as shown in Figure S2. In this system, a test solution was pumped into the microfluidic device by a syringe pump (LSP01-3A, Baoding Lange constant flow pump Co., Ltd., BaoDing, China). The injection scheme was designed as shown in Figure 4a. The test solution was injected into the sensor via an injection microchannel that was connected using a syringe with an amputated syringe needle and a flexible tube. Figure 4b shows the scheme for measuring the electrode admittance and transmitting it to a computer. Electrode admittance measurement was performed by AD5933-based circuits as shown in the dashed box, where a sinusoidal excitation signal was produced by the AD5933 chip. To minimize the influence of electrochemical reaction on admittance measurement, the highest frequency (100 kHz) and lowest amplitude (0.2 V) of the sinusoidal signal was set. Electrode polarization is a severe problem for admittance measurement, as it will generate microbubbles adsorbed on the electrode, which has a serious effect on the measurement results. So capacitance C2 was used to filter the DC component of the sinusoidal signal to avoid the DC polarization of the electrode. The design of the admittance measurement circuits in Figure 4b can completely avoid producing microbubbles and perform accurate admittance measurement based on the copper electrode. Detailed analysis about this is summarized in Part I of the Supplementary Materials. The real-time measured electrode admittance was read out by a microcontroller hat was integrated on a FPCB together with AD5933-based admittance measurement circuits. In an in vitro characterization experiment, the acquired real-time admittance information was transferred to a computer via a data-transfer circuit, while in the on-body trial, the information was temporarily stored in the microcontroller and was transferred to the computer by the data-transfer circuits after the sweat induction trial.

## 3. Results and Discussion

### 3.1. Characterizing the Sensing Performance of Calibration Area

**Figure 5:**
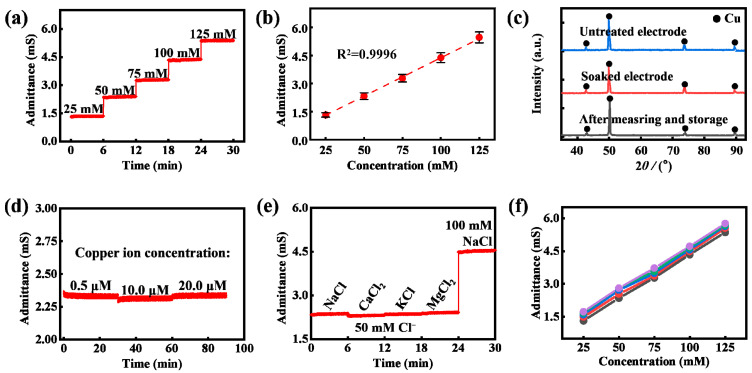
Sensing performance characterization of the calibration area. (**a**) Measured electrode admittances in NaCl solutions with different concentrations. (**b**) The calibration curve of the calibration area (N = 5), and the error bar represents standard deviation. (**c**) XRD characterization of copper electrodes. (**d**) Measured electrode admittances in 50 mM NaCl solutions with different copper ion concentrations (0.5, 10.0, and 20.0 μM). (**e**) Anti-interference performance of the calibration area. (**f**) Reproducibility of the sensor.

Since the sweat electrolyte concentration can affect the sweat-rate measurement, it should be simultaneously measured to calibrate the sweat rate according to Formula (2). In the calibration area, the model in Figure 2 can also be used to analyze the electrode admittance, which is only determined by the sweat electrolyte concentration when sweat completely saturates the electrode (referring to Part II of the Supplementary Materials). The sensing performance of the calibration area to the electrolyte concentration was characterized when NaCl solutions with different concentrations were injected into this area at the same rate of 2 μL·min^−1^. As displayed in Figure 5a, the measured electrode admittances stayed constant in the same NaCl concentration and increased with the NaCl concentration increasing. An approximate proportional relationship between the electrode admittance and the NaCl solution concentration was obtained (Figure 5b), which demonstrates that the conductance is dominant in the admittance measurement of the solution–electrode system. This is the key reason why copper electrodes can perform the measurement of sweat electrolyte concentration. X-ray diffraction (XRD) characterization was used to investigate whether the copper electrode was corroded during the experiment. The three electrode samples, respectively, were the untreated copper electrode, a copper electrode soaked in NaCl solution for 1 hour and saved for 1 week, and the electrode taken from the microfluidic patch that was used for the on-body trial, respectively. The characterization results of the XRD of the three copper electrodes are shown in Figure 5c, and the results reveal that there is no production of new substances after soaking and on-body measurement. It is due to that that the electrochemical transition between Cu atoms and Cu^2+^ is reversible and periodical when the excitation signal applied to the electrode is high-frequency sinusoidal AC voltage without the DC component. To further investigate the effect of copper ion concentration on the sensing performance of the sensor, the admittance measurements of 50 mM NaCl solutions with different copper ion concentrations were conducted, and the results in Figure 5d indicate that the change in copper ion concentration within sweat level has no significant effect on electrode admittance measurement, which benefits from a very low excitation voltage amplitude (0.2 V) and high excitation frequency (100 kHz) providing only weak drive force and short time for the electrochemical behavior of the copper ion. Besides, the copper ion level in sweat is very low (5–20 μM); therefore, very weak electrochemical behavior exists. Then, the calibration area was tested in the solutions with different types of cations but the same concentration of chloride ion, and the results in Figure 5e reveal that the major cations in sweat have no effect on the admittance-sensing performance of the calibration area. At last, five sensors in the same batch were used in the calibration test, and the results indicate that the sensor based on ultraviolet laser cutting has good reproducibility (Figure 5f).

Notice that chloride ions and bicarbonates are most abundant ions in sweat, while the concentration of chloride ions is approximately 20 times greater than that of bicarbonates in sweat [32]. Other sweat anions are derived mainly from the ionization of weak acids, including uric acid, lactate and ascorbic acid. Their ionization constants are 5.1 × 10^−6^, 1.4 × 10^−4^ and 5.0 × 10^−5^, respectively. So the concentrations of corresponding anions in sweat are far less than the chloride ion concentration due to their very low ionization constants. Thus, sweat conductivity is approximately proportional to the chloride ion concentration, just like in previous reports [21,33,34]. Therefore, for the on-body trial, the measured electrode admittance in the calibration area can not only be used to calibrate the measured sweat rate from the detection area, but it can also be converted to the sweat Cl^−^ concentration, which is also a critical parameter for the assessment of electrolyte balance and the diagnosis of cystic fibrosis [35].

### 3.2. Characterizing the Sensing Performance of Detection Area for Sweat Rate Measurement

**Figure 6:**
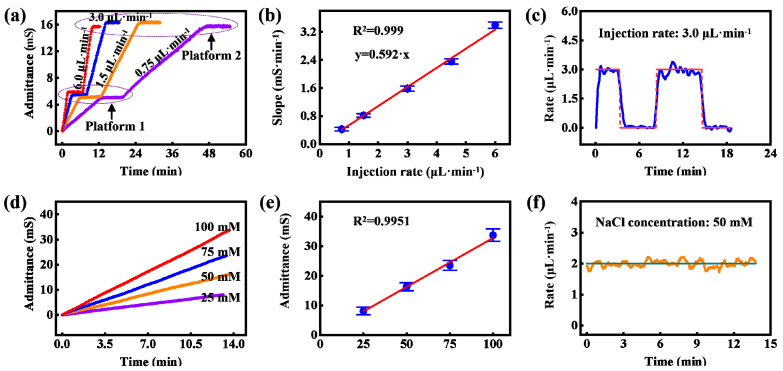
Characterizing the sensing performance of the detection area. (**a**) Measured electrode admittances under different injection rates but the same concentration of test solution. (**b**) The calibration curve between injection rate and the slope of increased admittance (N = 5), and the error bar represents standard deviation. (**c**) Measured flow rate at an injection rate of 3 μL∙min^−1^. (**d**) Measured electrode admittances under different concentrations of test solutions but the same injection rate. (**e**) The calibration curve between injection rate and electrode admittance when microchannel was filled with test solution (N = 5), and the error bar represents standard deviation. (**f**) Measured flow rate under 50 mM NaCl solution at the injection rate of 2 μL∙min^−1^.

To characterize the sensing performance of the detection area for sweat rate measurement, we first injected 50 mM NaCl test solutions into the detection area with different injection rates, and the injections were paused for a period of time when the test solutions arrived at a certain site in microchannels. During the pause, electrode admittances remained constant, as illustrated at Platform I in Figure 6a. When the solutions completely filled the microchannels, the admittance values were also unchanged, as illustrated at Platform II in Figure 6a. The two platforms indicate that there was no zero drift of our sensors, implying that the copper electrode can be used for sweat admittance measurement (more detailed analysis refers to Part I of the Supplementary Materials). Furthermore, the admittances of sweat with the same volume were approximately equal, as illustrated in Platform I and Platform II, demonstrating the reproducibility of the sensors. The change rates of the admittance with time in Figure 6a were proportional to injection rates (Figure 6b). This proportional relationship is consistent with Formula (2). As the experiments were conducted under the same rate, we could deduce the coefficient *K* in Formula (2), namely *K* = 50 mM/*k′* =84.5 mM·μL·mS^−1^, where *k′* is the slope of the fit curve in Figure 6b. Then we could obtain the measured flow rate according to Formula (2), as shown in Figure 6c and Figure S3. Then NaCl solutions with different concentrations were injected under the same rate, and the measured admittance changes are shown in Figure 6d. The admittance is approximately proportional to the solution concentration (Figure 6e), which validates the correctness of Formula (2). Figure 6f and Figure S4 show the measured sweat rates under different solution concentrations according to Formula (2). The experimental results indicate that the detection area combined with the calibration area is capable of accurately measuring the sweat rate.

**Figure 7:**
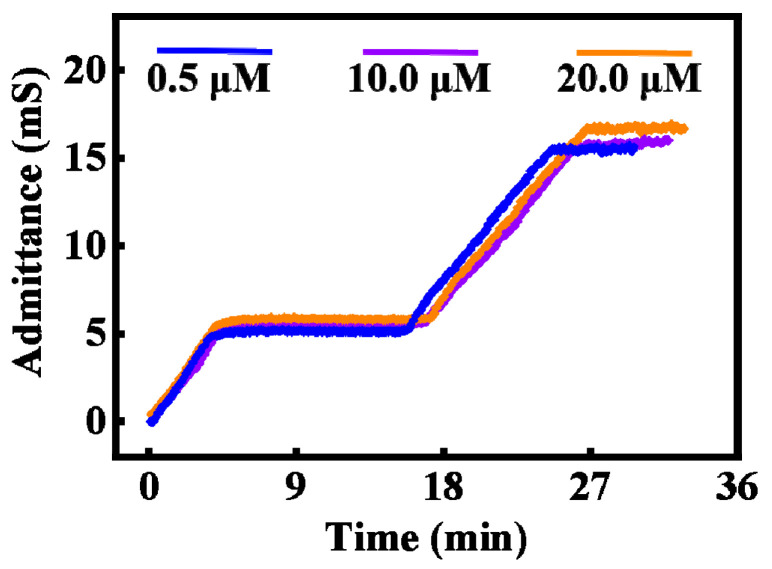
Measured electrode admittances of the detection area under 50 mM NaCl solutions with different copper ion concentrations (0.5, 10.0 and 20.0 μM) at the same injection rate.

As the copper electrode is utilized and the sweat copper-ion concentration ranges from 0.5 to 20 μM [32,36,37], it is necessary to investigate the influence of the copper ion concentration within sweat on the measurement result. Three 50 mM NaCl solutions containing different concentrations of copper ions were injected into the detection area, and the injections were paused at the same site of the microchannel for the same time. The results are displayed in Figure 7. The three curves almost overlap, which not only indicates the change in copper ion concentration within sweat has a negligible impact on electrode admittance measurement, but it also demonstrates the reproducibility of the sensor.

### 3.3. On-Body Trial of the Sweat Rate via the Microfluidic Patch

**Figure 8:**
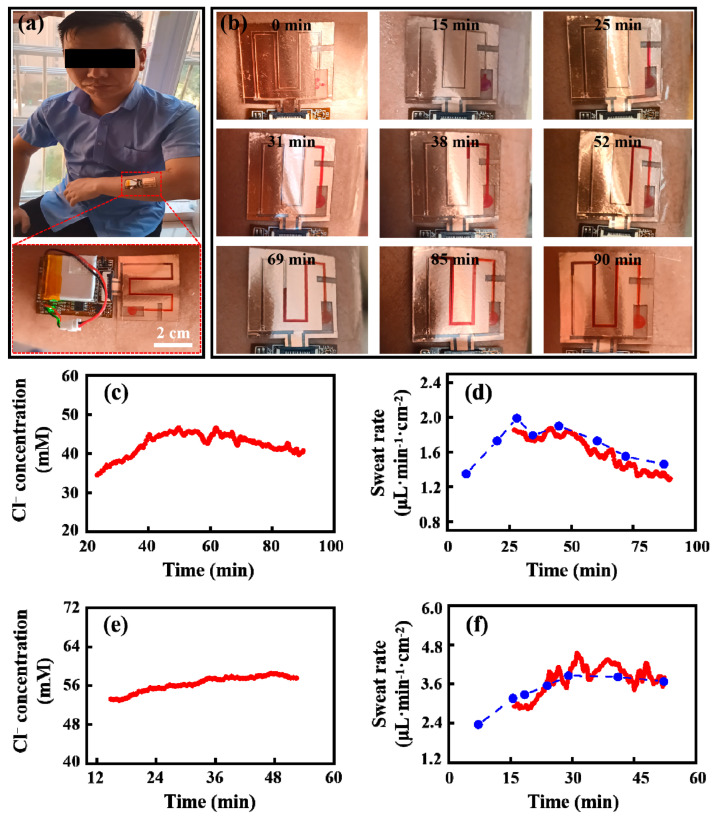
Results of on-body trial. (**a**) Depiction of on-body microfluidic patch and FPCB configuration mounted on the forearm of subject I during trial. (**b**) Photographs of sweat advancing in the microfluidic patch and corresponding time required to fill microchannel during the trial of subject I. (**c**) Measured sweat chloride ion concentration on the forearm of subject I from the admittance of the calibration area. (**d**) Measured sweat rate on the forearm of subject I from the admittance-based method and from the image-based method. (**e**) Measured sweat chloride ion concentration on the chest of subject II from the admittance of the calibration area. (**f**) Measured sweat rate on the chest of subject II from the admittance-based method and from the image-based method.

In on-body trial, the trials were conducted on three subjects (subject I, subject II, and subject III). The verification of the measurement accuracy of the sweat-rate sensor was conducted on the forearm of subject I and the chest of subject II, and the regional variations in sweat rate at different parts of the body was also studied on the chest, back, forearm, and forehead of subject III. All subjects had been given informed consent before the trials. Sweat induction was implemented by exposure to high temperatures for subject I and subject II and being exposed to high temperature and performing stationary biking for subject III. The real-time electrode admittances of the calibration area and the detection area were measured and saved by the FPCB (Figure 8a). Image-based sweat-rate measurement was used to verify the validity of the on-body sweat-rate measurement of the microfluidic patch during the trials of subject I and subject II. In details, the real sweat volume in the microchannel was acquired by recording the optical image of the dyed sweat. The sweat rate calculated from the change rate of image-based sweat volume can be used as a reference to assess the accuracy of the microfluidic sweat sensor, as in previously reported studies [5,14,16,38]. To highlight the captured sweat in the microchannel, a high-concentration dye solution (sudan red solution) was dropped into the collection area of the microfluidic patch before the patch adhered to the skin, then the water in solution was evaporated and only the Sudan red dye was left at the collection area [39]. During the sweat-collection trial, the dye will be redissolved and turn the sweat red in color. The optical images of the sweat in the microchannel are shown in Figure 8b for subject I and Figure S5 for subject II.

During the trial on the forearm of subject I, sweat filled the calibration area at 23.5 min, then the electrode admittance of this area could output the available sensing signal for the quantification of the equivalent chloride ion concentration. The chloride ion concentration measured from the calibration area is shown in Figure 8c, and it underwent a process of increasing and then decreasing during the whole trial. Sweat entered the detection area at 27.1 min, then the electrode admittance of this area was valid. For the trial on the chest of subject II, the measured chloride ion concentration is shown in Figure 8e, which increased slightly during the trial process. The sweat rate was calculated from the electrode admittance of the detection area and the measured sweat chloride ion concentration, according to Formula (2), and the result with the unit of μL∙min^−1^∙cm^−2^ is shown as the solid red line in Figure 8d,f for the two subjects when the sweat-collection area is considered. Besides, the referenced sweat rate could be obtained from the optical images of the dyed sweat, as the blue dots in Figure 8d,f. The measured sweat rate from the admittance-based method is consistent with the referenced one, demonstrating the validity of the on-body measurement of the proposed sweat-rate sensor.

From the referenced sweat rate, it can be found that sweat rate increased for a period of time after the onset of sweat induction, probably due to delayed perspiration from external stimuli. This trend is analogous to previously reported studies using other measuring approaches [13,14]. The change trend in the sweat rate is similar to that of the chloride ion, implying some correlations between the sweat chloride ion concentration and the sweat rate, which is consistent with previously reported studies [5,16,17]. This consistency may be because there is more time to reabsorb chloride ions with a low sweat rate and less time with a high sweat rate in the gland during the sweat secretion process [32].

**Figure 9:**
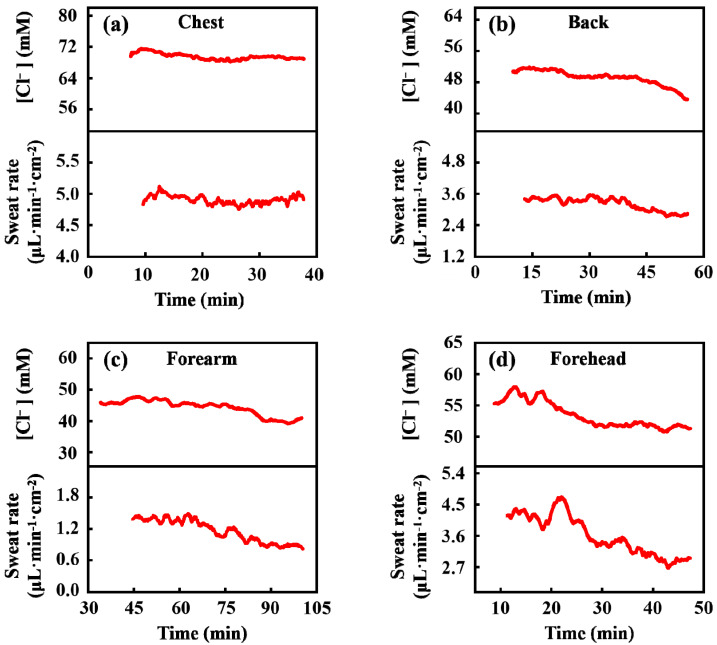
Regional sweat rate and chloride concentration at chest (**a**), back (**b**), forearm (**c**), and forehead (**d**) of Subject III, respectively.

In trials of different parts of the body of subject III, except for the forearm, all trials ended when the microchannels were filled. The results are illustrated in Figure 9. The sweat rate on his forearm is the lowest, while that on his chest is the highest. On the chest, the sweat rate almost stayed constant during the whole trial process, but on the other three parts, it declined especially in the second half of the trials. Comparing the results of the four parts, the sweat chloride ion concentrations were more concentrated at higher sweat rates, which is consistent with previous studies [17,40]. Furthermore, the change trend in sweat chloride ion is similar to that of the sweat rate. This trend is similar to previous reports [17,20], which may be due to the secretion rate of chloride ion in secretory coil of sweat glands increasing proportionally with the sweat rate, but it is larger than the reabsorption rate in the proximal duct of sweat glands, so that a higher final concentration of chloride ions is obtained at a greater sweat rate when sweat arrives at the skin surface [41].

Because the valid electrode admittance measured by FPCB only starts from sweat filling the calibration area or entering the detection area, there are delays for the measurements of sweat chloride ions and the sweat rate. Future studies should focus on the optimization of the structure of the microfluidic patch to reduce detection delay. Besides, the effective detection time of the sweat rate is limited by the volume of the microchannel. Notice that most studies indicate that the range of the sweat rate is generally ranges within 0.5 to 20 μL∙min^−1^∙cm^−2^ [5,9,12,13,15,16,42,43], as illustrated in Table S1. As the volume of the detection area is 22 μL, the effective detection time of the sweat-rate sensor approximately ranges from 5.5 min to 224.5 min for a 5 mm-diameter sweat-collection area in this work. The detection time can be extended by increasing the channel length or channel height so as to increase the volume of the microchannel.

## 4. Conclusions

To sum up, we proposed a simple, low-cost and easy-to-manufacture microfluidic patch for admittance-based sweat-rate sensing. This patch can be rapidly manufactured by only laser-cutting and transfer-printing, free of any surface treatment or modification of the electrodes and without alignment operation. A copper electrode made of copper foil tape was used for the measurement of the sweat rate. We successfully solved the potential interference problems of the copper electrode in admittance-based sweat-sensing by filtering the DC component of the excitation signal of the sensor and optimizing the frequency and amplitude of the signal. Compared with previously published studies, the copper electrode is capable of achieving the same performance as the gold electrode in the admittance-based measurement of sweat electrolyte concentration [17] and sweat rate [16]. However, under the condition of the same sensing performance, the proposed full-cut manufacture and design scheme can provide an easy-to-manufacture and low-cost path to fabricate a skin-interfaced sweat-rate sensor. Future studies will focus on improving the flexible circuits to enable wireless transmitting the measured data to mobile devices and optimizing the sensor structure to reduce detection delay.

## Data Availability

All relevant data is included in the manuscript and Supplementary Materials.

## Supplementary Materials

The following supporting information can be downloaded at: https://www.mdpi.com/article/10.3390/bios13010067/s1, PART I: The feasibility of copper electrode for admittance-based sensing of sweat rate [32,36,37]; PART II: Formula derivation of sweat rate [44]; PART III: Derivation process of the flow resistance of microchannel [7,45,46,47]; Part IV: Figure and table supplementary for detailing the design and characterization of the proposed microfluidic sweat-rate sensor; Figure S1: Layout of electrodes and centerline-gap in microchannel; Figure S2: Photograph of the in vitro flow injection test system; Figure S3: Measured flow rate at injection rate of 0.75, 1.5 and 6.0 μL∙min^−1^, respectively; Figure S4: Measured flow rate at the same injection rate of 2.0 μL∙min^−1^ under three test solutions with different NaCl concentrations; Figure S5: Photographs of sweat advancing in the microfluidic patch and corresponding time required to fill microchannel during the trial of subject II; Figure S6: EIS Nyquist plot of the electrodes in calibration area in different concentrations of chloride ion solutions; Table S1: The sweat rates in previously reported works.
