# Supplementary material for: Full-Cut Manufacture of Skin-Interfaced Microfluidic Patch with Copper Electrode for In Situ Admittance Sensing of Sweat Rate
Source: Biosensors (Basel). 2022 Dec 31;13(1):67. doi: 10.3390/bios13010067 (PMC9855928; doi:10.3390/bios13010067)
Supplement: Supplementary file 1 [file biosensors-13-00067-s001.zip › biosensors-2102912-supplementary.pdf]

biosensors

MDPI

Supplementary Materials

Full-Cut Manufacture of Skin-Interfaced Microfluidic Patch with Copper Electrode for In Situ Admittance Sensing of Sweat Rate

Lei Wei [1,2,†], Yuxin He [1,†], Zihan Lv [1], Daoyou Guo [1], Lin Cheng [1], Huaping Wu [3] and Aiping Liu [1,*]

[1] Key Laboratory of Optical Field Manipulation of Zhejiang Province, Zhejiang Sci-Tech University, Hangzhou 310018, China
[2] School of Physics and Electronics Engineering, Fuyang Normal University, Fuyang 236037, China
[3] Key Laboratory of Special Purpose Equipment and Advanced Processing Technology, Ministry of Education and Zhejiang Province, College of Mechanical Engineering, Zhejiang University of Technology, Hangzhou 310023, China
* Correspondence: liuaiping1979@gmail.com
† These authors contributed equally to this work.

PART I: The feasibility of copper electrode for admittance based sensing of sweat rate

A flexible gold electrode is the most commonly used electrode in electrochemical sensors, but it has the disadvantage of a high cost and a complex manufacturing process. By comparison, copper foil tape, a special tape with one adhesive side and another bare copper side, has the characteristics of flexibilit and, low cost and is easy to process by UV laser cutting. Furthermore, its inherent adhesion gives it the convenience of transfer printing. However, copper electrodes are not inert electrodes, so it is essential to investigate the feasibility for sweat admittance detection in this work.

Microbubbles adsorbed on an electrode are produced by hydrogen evolution and oxygen evolution reaction and will gradually reduce the measured electrode admittance with the increasing number of microbubbles, and it has a severe effect on measurement results. To determine whether some gas is produced on the surface of a copper electrode during the measurement process, we focus on the reduction potential of a possible electrode reaction, which is determined by the Nernst equation. At the cathode, the possible electrode reactions are $Cu^{2+}+2e^- = Cu$, and $2H^++2e^- = H_2$. Considering that the sweat copper ion concentration ranges from 0.5 to 20 μM [32, 36, 37] and that sweat pH is usually between 3 and 8, we can derive that the reduction potential of $E_{Cu^{2+}|Cu}$ ranges from 0.1504 to 0.1901V according to the Nernst equation and that the reduction potential of $E_{Cu^{2+}|Cu}$ ranges from -0.4863 to -0.1824V. In this case, $E_{Cu^{2+}|Cu}$ is larger than $E_{H^+|H_2}$, so $2H^++2e^-=H_2$ will not occur, and H_2 cannot be produced at the cathode. At the anode, we compare the reduction potential of the electrode reaction $Cu^{2+}+2e^- = Cu$, and $O_2+2H_2O+4e^- = 4OH^-$. For the latter, the reduction potential of $E^{\Theta}_{O_2|OH^-}$ ranges from 0.7656 to 1.0794V according to the Nernst equation, which is larger than that of $E_{Cu^{2+}|Cu}$. Furthermore, we set the amplitude of the excitation voltage of measurement circuits at 0.2 V, smaller than the reduction potential of $E^{\Theta}_{O_2|OH^-}$. So oxygen evolution will not occur at the anode.

To sum up, the electrochemical behavior at electrode interfaces is the periodically reversible transition between Cu atoms and Cu^{2+} instead of hydrogen evolution and the

oxygen evolution reaction. In order to minimize the effect of this electrode reaction on admittance measurement, we set the frequency of sinusoidal excitation in measurement circuits at a high value (100 kHz) and set its amplitude at a minimal value (0.2V), so that the electrochemical corrosion of electrode is negligible. From EIS experiment, we can prove that R_{ct} is far greater than X_c, implying that the electrochemical behavior during the measurement process is weak,and electrode impedance measurement is dominated by the sweat resistance that is proportional to sweat electrolyte concentration. This proportional relationship will be verified in the experiments in Fig. 5b, Fig. 6b and Fig. 6e. We can conclude that the copper electrode can be used for sweat admittance measurement.

PART II: Formula derivation of sweat rate

The equivalent circuit in Figure 2b can be further simplified as the admittance dY. Supposing that $Y(t)$ represents the added admittance of the newly filling sweat per unit length. Considering that flowing sweat in microchannel can be regarded as a series of parallel admittances between two electrodes (Figure 2a), the admittance dY can be written as

$$dY = Y(t)\cdot dl \quad . \tag{S1}$$

If A represents the section area of the microchannel, and $Q(t)$ represents the sweat rate with a unit of μL·min^{-1}, the volume of newly filling sweat during the time from t to $t+dt$ can be written as

$$dV = A\cdot dl = Q(t)\cdot dt \quad . \tag{S2}$$

Combining Formula (S1) and Formula (S2), we can obtain the sweat rate

$$Q(t) = \frac{A}{Y(t)}\cdot\frac{dY}{dt} = A\cdot Z(t)\cdot\frac{dY}{dt} \quad . \tag{S3}$$

Here $Z(t)$ is the reciprocal of $Y(t)$, representing the impedance of newly filling sweat into the detection area, whose magnitude is determined by the sweat electrolyte concentration. $Z(t)$ can be obtained by measuring the electrode admittance of calibration area. Furthermore, the double-layer capacitance is [44]

$$C_d = \frac{1}{\omega_B R_{ct}} \quad , \tag{S4}$$

where ω_B is the vertex of semicircle. We can get the capacitive reactance of C_d

$$X_c = \frac{1}{2\pi f_c C_d} \quad , \tag{S5}$$

where f_c is the frequency of sinusoidal excitation signal. Then we can obtain the parallel impedance of C_d, R_{ct} in Figure 2b

$$Z_p = \frac{\omega_B R_{ct}}{\omega_B + 2\pi f_c} \, . \tag{S6}$$

From Formula (S6), we can find that increasing f_c or decreasing R_{ct} can reduce Z_p. C_0 can be negligible due to the extremely thin electrode. We set the frequency of sinusoidal excitation signal to be its maximum value (100 kHz) and set the amplitude of the signal to be its minimum value (0.2 V) to minimize R_{ct}, so that Z_p will be minimized according to Formula (S6).

To study the magnitude of C_d and R_{ct} at the interface between the copper electrode and the electrolyte, the electrochemical impedance spectroscopy (EIS) was measured in a solution containing 20.0 μM copper ion and a different concentration of NaCl by an electrochemical workstation (CHI 660E, Shanghai Chenhua Instrument Co., Ltd, China), and the result is shown in Figure S6. From the fitting results of the EIS, it can be found that Z_p is far less than R_s. Therefore, $R_s(t)$ can approximately equal $Z(t)$ in Formula (S3), and Formula (S3) can be written as

$$Q(t) = A \cdot R_s(t) \cdot \frac{dY}{dt} \quad . \tag{S7}$$

Considering that the resistance of a dilute strong electrolyte solution is inversely proportional to the electrolyte concentration, Formula (S7) can be further written as

$$Q(t) = \frac{K}{c(t)} \cdot \frac{dY}{dt} \quad , \tag{S8}$$

where the coefficient K is determined by both the microchannel size and the layout of electrodes in the channel. It is a constant for a specific sensor. The differential form of Formula (S8) is

$$Q_i = \frac{K}{c_i} \cdot \frac{Y_i - Y_j}{t_i - t_j} \quad , \tag{S9}$$

where Q_i, c_i, and Y_i are the sweat rate, sweat electrolyte concentration and electrode admittance of detection area at the moment ti, respectively. t_i and t_j are two adjacent sampling moments.

PART III: Derivation process of the flow resistance of microchannel

When microfluidic device is attached to skin, sweat outflows from a sweat gland driven by the secretion pressure of the sweat gland and flows into a microchannel via the collection hole of the device. When the influence of capillary pressure is ignored, sweat flow rate in the rectangular microchannel is [7]

$$Q_{\mathrm{p}} = whP_s \frac{(\frac{wh}{w+h})^2}{8\mu L_s} \quad , \tag{S10}$$

where P_s is the residual pressure of sweat gland secretion pressure at the skin surface, w is the microchannel width, h is the microchannel height, L_s is the filled length of the microchannel, and μ is the sweat viscosity. So the flow resistance of the microchannel is

$$R_c = \frac{8\mu L_s}{wh}(\frac{1}{w} + \frac{1}{h})^2 . \tag{S11}$$

Because the cross section of microfluidic channel is micron in size, it is necessary to comprehensively consider the influence of the fluid capillary pressure on flow rate. The change in the flow velocity in the microchannel caused by capillary action is [45]

$$\Delta v = -\frac{C_g \Delta P_\sigma}{\mu L}, \tag{S12}$$

where C_g is the coefficient related to the channel size, which can be approximately expressed as [45]

$$C_g = \frac{1}{8}(\frac{wh}{w+h})^2 . \tag{S13}$$

Here ΔP_σ is the capillary pressure. For the rectangular microchannel, the capillary pressure can be expressed as [46, 47]

$$\Delta P_\sigma = -2\sigma(\frac{\cos\theta_1}{w} + \frac{\cos\theta_2}{h}), \tag{S14}$$

where σ is the surface tension coefficient of sweat, θ_1 is the contact angle between sweat and the sidewall of the microchannel, and θ_2 is the contact angle between sweat and the upper and lower surfaces of the microchannel. Bringing Formula (S13) and Formula (S14) into Formula (S12), the change of flow velocity in the microchannel caused by capillary action is

$$\Delta v = \frac{\sigma(\frac{\cos\theta_1}{w} + \frac{\cos\theta_2}{h})(\frac{wh}{w+h})^2}{4\mu L} . \tag{S15}$$

Then we can obtain the change of flow rate due to surface tension

$$\Delta Q = \Delta v \cdot S_{wh} = \frac{\sigma(\frac{\cos\theta_1}{w} + \frac{\cos\theta_2}{h})\frac{(wh)^3}{(w+h)^2}}{4\mu L}, \quad \text{(S16)}$$

where S_{wh} represents the cross-sectional area of the microchannel with width w and height h.

When considering the comprehensive effect of channel flow resistance and capillary pressure on sweat, the sweat flow rate is

$$Q = Q_P + \Delta Q = \frac{(wh)^3}{8\mu L(w+h)^2}\left[P_s + 2\sigma(\frac{\cos\theta_1}{w} + \frac{\cos\theta_2}{h})\right], \quad \text{(S17)}$$

where $P_s + 2\sigma(\frac{\cos\theta_1}{w} + \frac{\cos\theta_2}{h}) = P_s - \Delta P_\sigma$ represents the resultant pressure. Thus Formula (S17) can be written as

$$Q = \frac{P_s - \Delta P_\sigma}{R_c}. \quad \text{(S18)}$$

We can establish the sweat flow rate model, as illustrated in ModelI, according to Formula (S18).

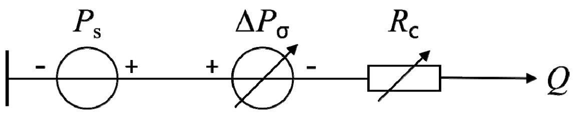

Model I: Equivalent physical mode of sweat flow rate, composed of P_s, ΔP_σ, R_c and Q.

Supposing that ΔP_σ is positive, ΔP_σ will reduce flow rate Q; in this case ΔP_σ equivalently increases the flow resistance of the microchannel. We can convert the effect of ΔP_σ to the flow resistance R_c, and the converted flow resistance of microchannel R_{eq} will be larger than R_c, namely

$$R_{eq} = R_c + \Delta R_\sigma, \quad \text{(S19)}$$

where ΔR_σ is the equivalent flow resistance derived from ΔP_σ.

Thus, ModelIcan be simplified as Model II.

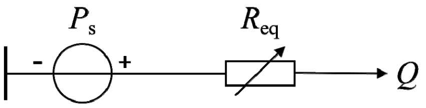

Model II: of the equivalent physical mode of sweat flow rate, composed of P_s, R_{eq} and Q.

Combining Model Iand Model II, we can obtain

$$\frac{P_s - \Delta P_\sigma}{R_c} = \frac{P_s}{R_{eq}}. \quad \text{(S20)}$$

The equivalent flow resistance can be written as

$$R_{eq} = \frac{P_s R_c}{P_s - \Delta P_\sigma}. \quad \text{(S21)}$$

Sweat gland secretion pressure produced at the secretion coil of gland drops along the dermal duct and the upper coiled duct of the gland during sweat secretion. Its residual pressure at the skin surface is P_s, which is the direct driving force to advance sweat in the microchannel. The magnitude of P_s is comprehensively determined by sweat gland secretion pressure, the pressure drop in the gland duct, and the external environment. When the sweat gland secretion pressure and flow resistance of the sweat gland duct are taken into account, the sweat flow rate model can be further expressed as Model III.

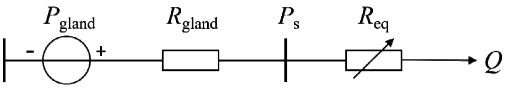

Model III: of the equivalent physical mode of sweat flow rate, composed of P_{gland}, P_s, R_{gland}, R_{eq} and Q.

Here P_{gland} is the sweat gland secretion pressure, and R_{gland} is the flow resistance of the sweat gland duct. In this case, the flow rate Q can be written as

$$Q = \frac{P_{gland}}{R_{gland} + R_{eq}} \quad . \tag{S22}$$

The equivalent flow resistance R_{eq} can be written as

$$P_s = Q \cdot R_{eq} = R_{eq} \frac{P_{gland}}{R_{gland} + R_{eq}} \quad . \tag{S23}$$

Combining Formula (S22) and Formula (S23), we can obtain P_s

$$P_s = \frac{P_{gland} R_c + \Delta P_\sigma R_{gland}}{R_c + R_{gland}} \quad . \tag{S24}$$

Bringing Formula (S24) into Formula (S21), we can obtain the equivalent flow resistance

$$R_{eq} = \frac{P_{gland} R_c + \Delta P_\sigma R_{gland}}{P_{gland} - \Delta P_\sigma} \quad . \tag{S25}$$

Part IV: Figure and table supplementary for detailing the design and characterization of the proposed microfluidic sweat rate sensor

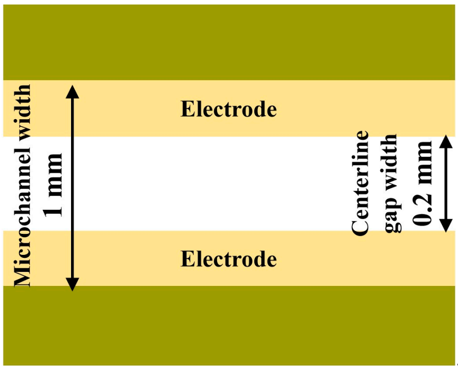

Figure S1. Layout of electrodes and centerline-gap in microchannel.

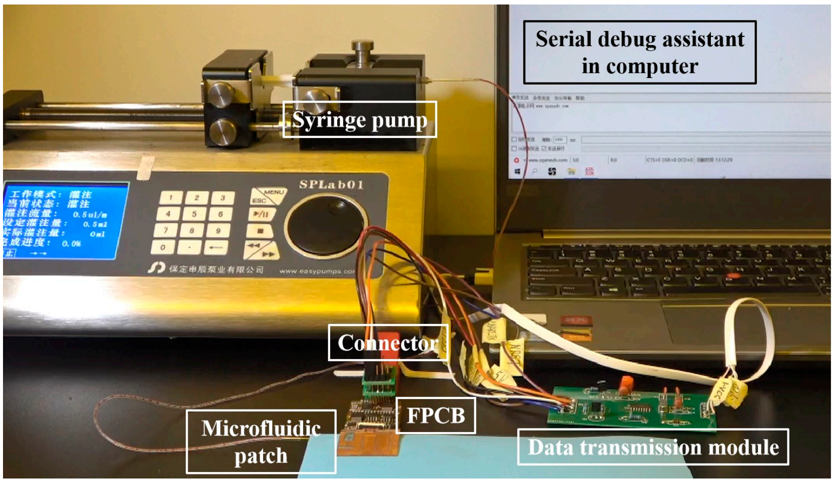

Figure S2. Photograph of the in vitro flow injection test system.

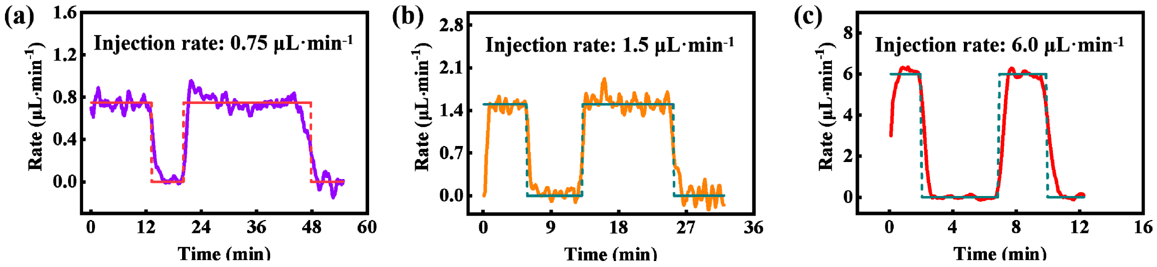

Figure S3. Measured flow rate at injection rates of 0.75, 1.5 and 6.0 μL·min^{-1}, respectively.

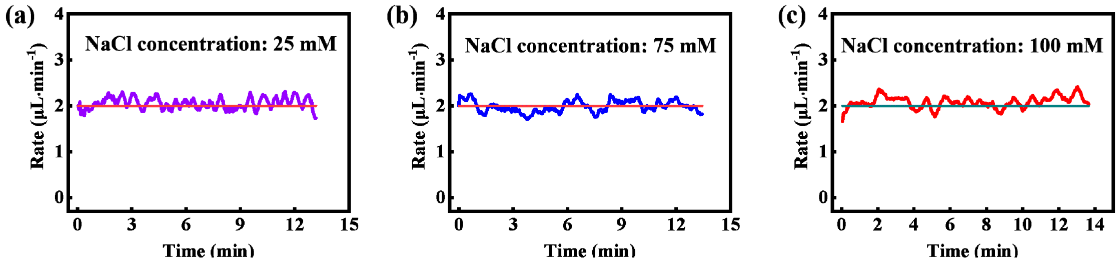

Figure S4. Measured flow rate at the same injection rate of 2.0 μL·min^{-1} under three different NaCl concentrations (25 Mm, 50 mM and 100 mM).

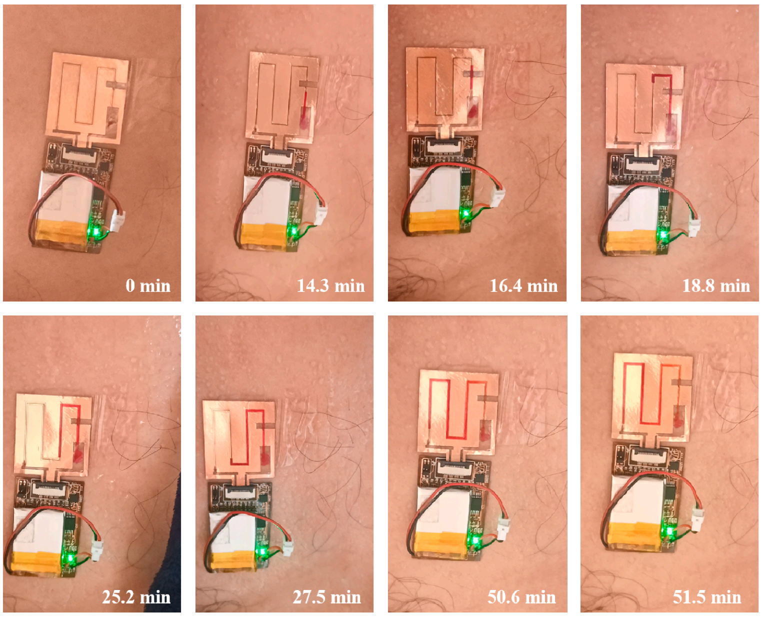

Figure S5. Photographs of sweat advancing in the microfluidic patch and corresponding time required to fill microchannel during the trial of subject II.

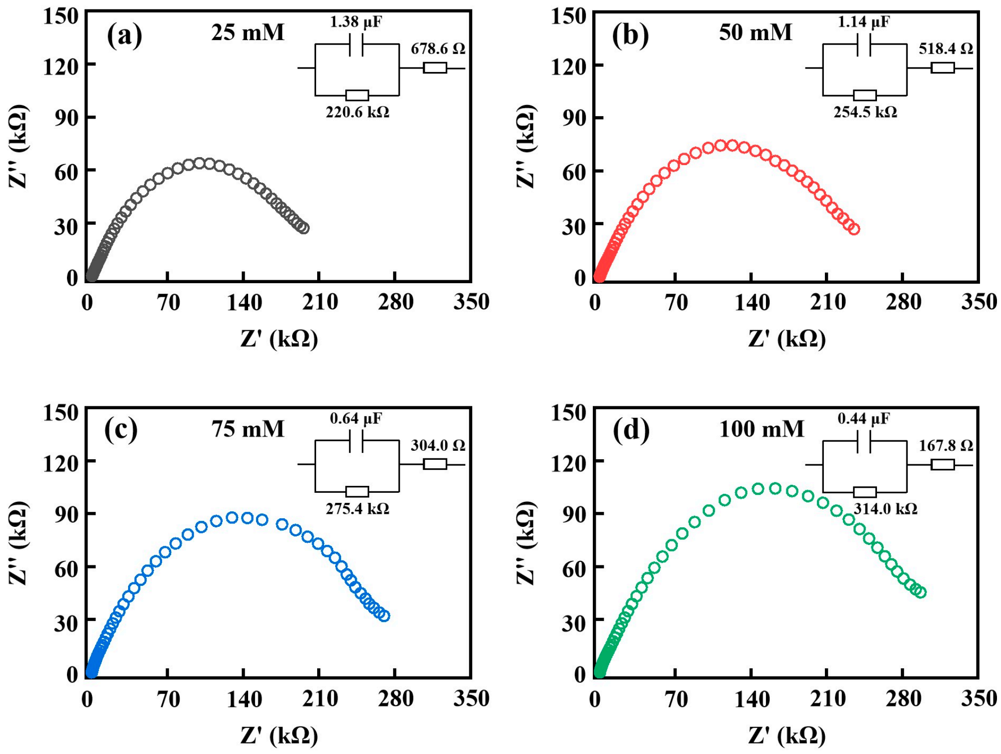

Figure S6. EIS Nyquist plot of the electrodes in calibration area in different concentrations of chloride ion solutions.

EIS measurements of the electrodes in the calibration area is carried out with different concentrations of chloride ion solutions (25 mM, 50 mM, 75 mM, 100 mM) with a constant copper ion concentration of 20 μM. The results are as below. The corresponding equivalent circuit of each EIS curve shown in subgraph was obtained by fitting the EIS data in electrochemical impedance spectrum fitting software (ZView). From the equivalent circuits, it could be found that the parallel impedance of double-layer capacitance and charge-transfer resistance ($X_c // R_{ct}$) is far less than solution resistance (R_s) when the frequency of sinusoidal excitation is set to 100 kHz; in this case, the electrode admittance is

$$Y = \frac{1}{Z_p} = \frac{1}{(\mathrm{Xc} // \mathrm{Rct}) + R_s} \approx \frac{1}{R_s} = G_s$$

Therefore, the electrode admittance (Y) is approximately proportional to the sweat electrolyte concentration.

Table S1. The sweat rates in previously reported works.

Sweat rate ($\mu L \cdot min^{-1} \cdot cm^{-2}$)	Ref.	Sweat rate ($\mu L \cdot min^{-1} \cdot cm^{-2}$)	Ref.
0.5-6.0	*5*	8.0	*15*
1.0-2.0	*9*	1.0-3.6	*16*
1.6	*12*	0.6-9.0	*35*
7.5-20.0	*13*	1.6-5.0	*36*

Reference

R1. Baker, L.B.; Wolfe, A.S. Physiological mechanisms determining eccrine sweat composition, Eur. J. Appl. Physiol. **2020**, 120, 719-752.

R2. Bagheri, N.; Mazzaracchio, V.; Cinti,S.; Colozza, N.; Natale, C.D.; Netti, P.A.; Saraji, M.; Roggero, S.; Moscone, D.; Arduini, F. Electroanalytical sensor based on gold-nanoparticle-decorated paper for sensitive detection of copper ions in sweat and serum, Anal. Chem. **2021**, 93, 5225-5233.

R3. W. Gao, H.Y.Y. Nyein, Z. Shahpar, H.M. Fahad, K. Chen, S. Emaminejad, Y. Gao, L.C. Tai, H. Ota, E. Wu, J. Bullock, Y. Zeng, D.H. Lien, A. Javey, Wearable microsensor array for multiplexed heavy metal monitoring of body fluids, ACS Sens. **2016**, 1, 866−874.

R4. Randviir, E.P.; Banks, C.E. Electrochemical impedance spectroscopy: an overview of bioanalytical applications. Anal. Methods **2013**, 5, 1098-1115.

R5. Reeder, J.T.; Choi, J.; Xue, Y.; Gutruf, P.; Hanson, J.; Liu, M.; Ray, T.; Bandodkar, A.J.; Avila, R.; Xia, W.; Krishnan, S.; Xu, S.; Barnes, K.; Pahnke, M.; Ghaffari, R.; Huang, Y.; Rogers, J.A. Waterproof, electronics-enabled, epidermal microfluidic devices for sweat collection, biomarker analysis, and thermography in aquatic settings. Sci. Adv. **2019**, 5, eaau6356

R6. Delamarche, E.; Bernard, A.; Schmid, H.; Bietsch, A.; Michel, B.; Biebuyck, H.; Microfluidic networks for chemical patterning of substrates: design and application to bioassays. J. Am. Chem. Soc. **1998**, 120, 500-508.

R7. Choi, J.; Kang, D.; Han, S.; Kim, S.B.; Rogers, J.A. Thin, soft, skin-mounted microfluidic networks with capillary bursting valves for chrono-sampling of sweat. Adv. Healthc. Mater. **2017**, 1601355.

R8. Ladosz, A.; von Rohr, P.R.; Design rules for microscale capillary phase separators, Microfluid. Nanofluid. **2017**, 21, 153.
